# Reducing physical restraints by older adults in home care: development of an evidence-based guideline

**DOI:** 10.1186/s12877-020-1499-y

**Published:** 2020-05-07

**Authors:** Kristien Scheepmans, Bernadette Dierckx de Casterlé, Louis Paquay, Hendrik Van Gansbeke, Koen Milisen

**Affiliations:** 1 Wit-Gele Kruis van Vlaanderen, Nursing Department, Frontispiesstraat 8, bus 1.2, 1000 Brussels, Belgium; 2 Department of Public Health and Primary Care, Academic Centre for Nursing and Midwifery, KU Leuven, Kapucijnenvoer 35 blok d – bus 7001, B-3000 Leuven, Belgium; 3 Division of Geriatric Medicine, Department of Internal Medicine, Leuven University Hospitals, Herestraat 49, 3000 Leuven, Belgium

**Keywords:** Evidence based, Home care, Physical restraints, Practice guideline, Reduction, Nurses, Nursing

## Abstract

**Background:**

Restraint use is a complex and challenging issue in home care. Due to socio-demographic trends, worldwide home healthcare providers are faced with an increasing demand for restraint use from informal caregivers, patients and healthcare providers, resulting in the use of various types of restraints in home care. Awareness and knowledge of restraint use in home care, its implications and the ethical challenges surrounding it are of crucial importance to its reduction. This research aimed to describe the development process of an evidence-based practice guideline to support caregivers to optimize home care.

**Method:**

The practice guideline was developed according to the framework of the Belgian Centre for Evidence-Based Medicine and AGREE II. The guideline was developed over several stages: (1) determination of the target population and scope, (2) literature search, (3) drafting and (4) validation. A multidisciplinary working group determined the proposed purpose, target group, and six clinical questions for the guideline. A consensus procedure and consultation by experts were used to develop the guideline.

**Results:**

The guideline provides an answer to six clinical questions and contains ten key recommendations based on the classification of GRADE, with the objective of increasing healthcare providers’ awareness, knowledge and competence to adequately deal with situations or questions related to restraint use. The guideline also includes a flowchart for dealing with complex situations where the use of restraints is requested, already present or considered.

**Conclusions:**

The guideline was validated by the Belgian Centre for Evidence-Based Medicine. Increasing competence, awareness and knowledge related to restraint use are key objectives of the guideline for reducing restraint use in home care. A multicomponent intervention to support healthcare workers in implementing the guideline in clinical practice needs to be developed.

## Background

Research indicates that, depending on the definition used, the prevalence of restraint use in home care varies between 7% [1], 9.9% [2] and 24.7% [3, 4]. The prevalence of physical restraint use among older adults with cognitive impairment in the home is 38% (95% CI 35–42) [5]. Due to recent demographic changes and technological innovations, there is an increasing number of frail older persons living at home, resulting in a growing demand for home care [6]. The balance in long-term care service provision has tended to shift towards home-based care because most older people prefer home care to institutionalization and because home care is more cost-effective [7–9]. As a consequence, healthcare providers are faced with an increasing demand from, e.g., informal caregivers, for restraint use in the home [4].

Experiences from daily practice and the limited literature on restraint use in home care strongly suggest that healthcare providers (e.g., nurses) are insufficiently aware of the meaning and content of the existing concepts related to restraint use (i.e., restraints, physical restraints), resulting in restraint use in clinical practice and its subsequent negative impact on patients [10]. Patient safety is the most commonly indicated reason for using (physical) restraints in the home care setting, according to healthcare professionals [3, 4, 9–11]. Yet this finding is in contrast to evidence from nursing homes that restraint use does not protect patients but, on the contrary, enhances the risk of various physical problems (e.g., physical harm, pressure ulcers, and injurious falls) [12]. This lack of awareness is compounded by uncertainties in legislation and the (ethical and legal) responsibilities of healthcare providers and informal caregivers in regard to safe and respectful care; furthermore, nurses are often unaware of effective interventions to meet patient needs in challenging situations [9, 10, 13].

Decision-making related to restraint use is a complex process that is influenced by various factors. Research from nursing homes indicates that not only patient characteristics such as cognitive decline and poor mobility but also nonpatient-related factors such as the attitude and knowledge of healthcare providers and legislation affect decision-making about restraint use [12, 14–19]. The context-specific factors influencing restraint use include insufficient supervision, decreases in wellbeing of informal caregiver and dissatisfaction with family support [11]. Legislation and/or regulations may limit the use of restraints in some settings. In Belgium, however, the legal framework is inadequate to provide clear guidance for clinical practice in home care [9]. A clear policy within home care organizations and guidelines to deal with restraint use in home care are lacking [10, 13, 20].

Being exposed to decisions and practice related to restraint use has a considerable impact on nurses, as illustrated in the study of Scheepmans et al. [10]. Nurses underline the complexity of deciding whether to use constraints because of the opposing needs and various interests of the actors involved (e.g., between the nurse and informal caregiver and between the patient and informal caregiver) [9]. This may explain nurses’ experience of moral distress when confronted with restraint demands or use [21].

Considering the complexity of restraint use in home care [9], healthcare providers need to increase their awareness and knowledge of the issue to improve their decision-making. Therefore, we aimed to describe the development process of a practice guideline to support healthcare workers in preventing and reducing the use of physical restraints in home care.

## Method

The practice guideline was developed according to the framework of the Belgian Centre for Evidence-Based Medicine (CEBAM) and the Appraisal of Guidelines for Research & Evaluation II (AGREE II) [22]. The guideline was developed by the research team together with a representative multidisciplinary working group and comprised four different stages: (1) determination of the target and user populations and scope, (2) literature search, (3) drafting and (4) validation.

### Stage 1: Determination of the target and user populations and scope

The target patient population was determined as home-dwelling persons, aged 60 or older, in home care and with an increased risk for physical restraint use (e.g. cognitive impairment, poor mobility). The decision to focus on this specific target population was made in collaboration with a specially constructed multidisciplinary working group based on the expertise of the members of the working group, the state of the art of the current literature (home care and residential care in older persons) and the results of a qualitative study on the experiences of home nurses regarding restraint use in older persons in home care in Flanders (Belgium) [10].

The target users of the guideline were healthcare providers in home care (e.g., home nurses, nursing aides, domestic aides, general practitioners, occupational therapists and physiotherapists).

Considering the lack of knowledge of and available evidence on restraint use in home care and based on the experience of the multidisciplinary working group, six clinical questions were formulated based on consensus among the members of the working group to define the guideline’s scope: (1) What is meant by physical restraint use in home care? (2) What factors increase the risk of physical restraint use in home care? (3) What are the consequences and impact of physical restraint use in home care? (4) What ethical and legal framework can support healthcare providers in decisions about the use of physical restraint in home care? (5) How can healthcare workers reduce physical restraint use in home care? (6) What steps and persons need to be involved in the decision-making process regarding and application of physical restraints in home care? The first five questions aim to increase awareness and knowledge of the problem of restraint use in home care. These insights and knowledge are required to understand the flowchart and how to deal with complex situations where the use of restraints is considered, requested or already present (question 6).

The research team recruited a multidisciplinary working group (*n* = 7) comprising home nurses (*n* = 2), domestic aides (*n* = 2), a general practitioner (*n* = 1) and representatives of patients and informal caregivers (*n* = 2). Members were selected to match the expertise of the research team (*n* = 4), consisting of persons with expertise in home care (*n* = 2); physical restraints, delirium, and falls (*n* = 1); and the ethics of care (*n* = 1). In addition, 2 lawyers and an ethicist were consulted to explain the legal and ethical frameworks for restraint use in home care.

### Stage 2: Literature search

The availability of existing national and international guidelines on physical restraint use in home care was determined by conducting a literature search. Because of the absence of available guidelines in this setting, the search was expanded to include research involving residential settings. Publications were considered if they met the following criteria: (1) reporting on older persons, restraint use, chronic care and (practice) guidelines and (2) written in English, French or Dutch. Guidelines on restraint use related to children; schools; psychiatry; seclusion; acute, emergency or intensive care; or dentistry were excluded. Five databases (i.e., PubMed, Embase, Psych Info, Cinahl and Invert) and online (inter)national guideline databases (including New Zealand Guidelines Groups, National Clearinghouse, Guideline Finder UK, SIGN [Scottish Intercollegiate Guidelines Network], NICE [National Institute for Health and Care Excellence], World Health Organization [WHO] guidelines, Canadian Medical Association InfoBase Clinical Practice Guidelines, Haut Autorité de Santé, Agency for Healthcare Research and Quality, KCE [Federaal kenniscentrum voor Gezondheidszorg], Domus Medica and ‘Société Scientifique de Médecine Générale’) were searched from March 2013. Five guidelines [23–28] were found and assessed using AGREE II (Table 1). Based on the AGREE II results, two of them were retained [23, 28]. A third guideline [24] scored almost equally well on the quality assessment as the clinical guideline of Milisen et al. [28]. Because the content of the RCN guideline was based mainly on legislation in the UK, which was not the primary focus of the study and not applicable to the Belgian context, we excluded this guideline.

**Table 1:** Quality appraisal of existing guidelines according to Agree II

	JBI, 2002 (Pt 1 & 2) [25, 26]	Irish Nurses Organisation, 2003 [27]	Anaes, 2000 [23]	Royal College of Nursing, 2008 [24]	Milisen et al., 2006 [28]
**Domain 1: Scope and Purpose**					
1. The overall objective(s) of the guideline is (are) specifically described.	5	7	7	7	7
2. The health question(s) covered by the guideline is (are) specifically described.	4	2	6	5	4
3. The population (patients, public, etc.) to whom the guideline is meant to apply is specifically described.	3	3	6	7	7
Subtotal	12	12	19	19	18
**Domain 2: Stakeholder Involvement**					
4. The guideline development group includes individuals from all the relevant professional groups.	4	2	6	6	6
5. The views and preferences of the target population (patients, public, etc.) have been sought.	2	1	4	3	3
6. The target users of the guideline are clearly defined.	2	4	5	5	4
Subtotal	8	7	15	14	13
**Domain 3: Methodology**					
7. Systematic methods were used to search for evidence.	1	1	5	1	1
8. The criteria for selecting the evidence are clearly described.	1	1	1	1	1
9. The strengths and limitations of the body of evidence are clearly described.	1	1	2	1	1
10. The methods for formulating the recommendations are clearly described.	1	1	1	1	1
11. The health benefits, side effects and risks have been considered in formulating the recommendations.	3	2	2	2	2
12. There is an explicit link between the recommendations and the supporting evidence.	2	3	5	3	3
13. The guideline has been externally reviewed by experts prior to its publication.	5	1	6	3	2
14. A procedure for updating the guideline is provided.	1	1	1	1	1
Subtotal	15	11	23	13	12
**Domain 4: Clarity of Presentation**					
15. The recommendations are specific and unambiguous.	6	6	6	5	7
16. The different options for management of the condition or health issue are clearly presented.	4	3	3	4	4
17. Key recommendations are easily identifiable.	5	3	3	5	6
18. The guideline describes facilitators and barriers to its application.	1	1	1	1	1
Subtotal	16	13	13	15	18
**Domain 5: Applicability**					
19. The guideline provides advice and/or tools on how the recommendations can be put into practice.	3	5	6	3	4
20. The potential resource implications of applying the recommendations have been considered.	1	1	1	1	1
21. The guideline presents monitoring and/ or auditing criteria.	1	1	1	1	1
Subtotal	5	7	8	5	6
**Domain 6: Editorial Independence**					
22. The views of the funding body have not influenced the content of the guideline.	3	1	1	1	1
23. Competing interests of guideline development group members have been recorded and addressed.	2	1	1	1	1
Subtotal	5	2	2	2	2
**Overall guideline assessment**					
- Rate the overall quality of this guideline: 1 (lowest possible quality) – 7 (highest possible quality)	3	4	5	4	5
- I would recommend this guideline for use					
◦ Yes					
◦ Yes, with modifications	+	+	+	+	+
◦ No					
Notes		Implemen-tation schedule (+)	Overview by behaviour and scores of alternatives	Legislation of UK (−) Examples for clarification (+) Employers involved (+) Ethical aspects (+)	Belgian context flowchart
Total	61	52	80	68	69

The quality appraisal according to Agree II consists of 23 items divided over 6 domains. Each item is rated on a 7-point scale ranging from strongly disagree (1) to strongly agree (7). The assessment is based on the total score of the 23 items and whether the user wants to recommend the guideline for use. Because there is not a set of minimum scores to judge the quality, the decision is made by the user and the context in which AGREE II is used [22]

Next, a literature search (July 2015) focused on the aforementioned six predetermined clinical practice questions. For each question, the literature search consisted of two phases. First, the articles were checked for relevance to the topic of home care, and articles of any design written in English, Dutch or French were eligible for inclusion. Second, this review was supplemented by a search of review articles related to the residential setting published in the last 5 years, with the same language criteria as for home care.

PubMed and Cinahl were the databases consulted for both searches. For only the first clinical question in the home care setting, an additional database (i.e., Embase) was consulted. To construct the search string, medical subject headings were combined with free search terms using Boolean operators (AND / OR). The same groups of search terms (i.e., restraints and aged) were used for both searches and combined with search terms in function of the subject of the clinical question and setting. The search string of the home care setting was completed with variations on the term ‘home care’. For the residential setting, combinations of key words for review were added (see Additional file 1: overview of all search strategies per clinical question (home care and residential setting)).

One author (KS) conducted the guideline and literature search, removed duplicate publications and made first selection of articles based on titles and abstracts. The methodological quality of the articles was assessed by two authors (KS, LP). Differences between their scores were discussed within the research team.

Various tools were used to assess the quality of the articles according to the study design: VAKS (Danish acronym for Appraisal of Qualitative Studies) [29], MINORS (Methodological Index for Non-Randomized Studies) [30] and AMSTAR (Assessing the Methodological Quality of Systematic Reviews) [31]. Table 2 gives an overview of the retained articles and the quality assessment by clinical question and setting. Using the snowball method, reference lists were checked, which resulted in the inclusion of three additional articles [14, 39, 40]. Four articles [14, 39, 41, 42] and one chapter from the book of Gastmans and Vanlaere [43] contained important background information drawing upon expert opinion while not reporting primary research. As a consequence, there was no quality assessment for those items.

**Table 2:** Overview of articles by clinical question, setting and quality appraisal

Author	Setting	Type	Clinical Practice Question Addressed	Quality appraisal		
				AMSTAR^a^	VAKS^b^	MINORS^c^
Beerens et al., 2014 [2]	HC	Survey	1			10/10
de Veer et al., 2009 [13]	HC	Survey	1			9/10
Evans and FitzGerald, 2002 [40]	Res	Review	2	6/10		
Evans and Cotter, 2008 [39]	HC	Paper	5, 6	NA	NA	NA
Evans et al., 2003 [46]	Res	Review	3	4.5/10		
Gastmans and Milisen, 2006 [14]	Res	Paper	1,2, 4–6	NA	NA	NA
Gastmans and Vanlaere, 2005 [43]	Res	Book	2–4	NA	NA	NA
Goethals et al., 2012 [15]	Res	Review	2–4	6/10		
Goethals et al., 2013 [35]	Res	Qualitative study	4		14.6	
Hamers and Huizing, 2005 [41]	HC, Res	Paper	1–3	NA	NA	NA
Hellwig, 2000 [42]	HC	Paper	1, 3, 5, 6	NA	NA	NA
Hofmann and Hahn, 2014 [12]	Res	Review	1–3	6/10		
Köpke et al., 2012 [51]	Res	RCT	5, 6			10/10
Kurata and Ojima, 2014 [48]	HC	Survey	1			9/10
Lane and Harrington, 2011 [49]	Res	Review	1	5/10		
Möhler et al., 2012 [52]	Res, HC	Review	1, 5, 6	7/10		
Möhler et al., 2014 [19]	Res	Review	1, 3, 4	7/10		
Scheepmans et al., 2014 [10]	HC	Qualitative study	1		15	

*NA* not applicable, *HC* home care, *Res* residential

^a^Shea et al., 2007. AMSTAR is a validated instrument and consists of 11 items with 4 answer possibilities [31]

^b^The assessment tool VAKS consists of 30 questions related to 5 criteria (i.e. formal requirements, credibility, transferability, dependability and confirmability). Based on the total score an article is ‘recommended’ (≥ 15), ‘recommended with reservations’ (≥ 10 < 15) or ‘not recommended’ (< 10) [29]

^c^The MINORS consists of 12 items, the first 8 items are for non-comparable studies. The scores for the individual items can be 0 (not reported), 1 (reported but inadequate) or 2 (reported and adequate) [30]

### Stage 3: Development of the practice guideline

The literature search confirmed that no guidelines specific to restraint use in home care and that research on restraint use in home care settings was scarce. For this reason, the multidisciplinary working group used a consensus procedure to develop the practice guideline [32, 44]. Our consensus method existed of different steps. First, once the clinical practice questions were decided by the working group, one member of the research team prepared the responses to the different questions based on the literature search. Second, the content was first discussed by the entire research team. Third, the adapted version was presented to and discussed with the multidisciplinary working group. Special attention was given to translate the residential setting literature to the home care setting. Feedback was used by the members of the research team to refine the content. The adapted text was then again discussed with the multidisciplinary working group. This process was repeated until consensus was reached.

The development of the practice guideline resulted in ten recommendations in response to the six clinical practice questions and a flowchart to support the decision-making process when the use of physical restraints is requested or considered. To indicate the quality of evidence and strength of the recommendations, we used the methodology based on GRADE (Grading of Recommendations Assessment, Development and Evaluation system), which was required by the validation committee [34].

A preliminary version of the guideline was read by the organizations to which the members of the multidisciplinary working group belonged. They checked whether the content of the guideline was applicable to their organization. The guideline was revised based on the experience of and feedback from these organizations. Furthermore, the revised version of the guideline was discussed with the multidisciplinary working group using clinical cases. Based on this feedback, a penultimate version was developed by the research team and presented to and discussed with a group of clinical practitioners (i.e., nurses, occupational therapists, general practitioners, and physiotherapists). The text was again adapted one last time.

### Stage 4: Validation of the practice guideline

The resultant practice guideline was presented to the Belgian Centre for Evidence-Based Medicine (CEBAM) for validation. The aim of this independent validation was to guarantee the methodological quality of the guideline. A validation committee was formed consisting of one methodological expert, one content expert, a chair (president of CEBAM and a general practitioner) and three experienced clinicians representing the disciplines for which the guideline was developed (one home care nurse, one occupational therapist, and one general practitioner). The validators assessed the guideline using the AGREE II instrument [22], and they discussed the criteria for which the validators had divergent scores. Next, the validation committee came to a final judgement, for which there were three possible final decisions: the guideline is validated as such, possibly with minor comments (1); the guideline is validated, provided that the authors meet the major comments (2); or the guideline cannot be validated in its current state (3). Next, the validation committee discussed all their remarks with the researchers (KS, KM). The most important comments related to the focus of the guideline (application rather than prevention of restraints in home care), some aspects of the method (more specification requested) and the recommendations (clear delimitations of the key recommendations). The commission decided that the guideline could be validated provided that the major comments were addressed. The guideline was again revised by the research team in response to the comments of the validation commission and finally approved in its current form by CEBAM on 15 December 2015.

## Results

The current article provides only a summary of the results. The full guideline has been published in book format [33] and a summary of the guideline is available on the website www.fixatiearmethuiszorg.be. The practice guideline answers six clinical questions, makes 10 recommendations (Table 3), and is accompanied by a flowchart that illustrates the steps to be taken and the persons to involve in the decision-making process for the application of physical restraints in home care (sixth question).

**Table 3:** Overview of the six clinical questions, ten recommendations, quality of the evidence and strength of the recommendations according to GRADE

Clinical Practice Question	No.	Recommendations	GRADE
1. What is meant by physical restraint use in home care?	1.	A definition of physical restraint should be used in home care. The following definition is suggested: as ‘Physical restraint is any device, material or equipment, attached to or near a person’s body and which cannot be controlled or easily removed by the person and which (is) deliberately (intended to) prevent(s) a person’s free body movement to a position of choice and\or a person’s normal access to their body’ (Retsas, 1998). (e.g. bedrails, bed-against-the-wall (positioned in a way that the person will not fall out of bed), locked room or house doors, deep chair that prevents rising and restrictive clotting and belts).	1 C
	2.	Healthcare providers should be aware that the application of any measure that limits free movement of the patient, regardless of its purpose, is a form of restraints.	1 C
2. What factors affect the probability of physical restraint use in home care?	3.	Healthcare providers should take the complex set of risk factors into account that affect the probability of physical restraint use in home care:	1 B
		- personal (e.g., poor mobility) and contextual factors	
		- knowledge and attitudes of healthcare providers	
		- culture of home care organisation	
		- legislation	
3. What are the consequences and the impact of physical restraint use in home care?	4.	The use of physical restraints should be avoided as much as possible due to the negative physical and psychosocial consequences for the patient.	1 A
	5.	Healthcare providers should be aware of the negative impact of physical restraints on the informal caregiver and should pay attention to support them.	1 C
	6.	Healthcare organizations should be aware of the impact of using physical restraints on the involved healthcare providers.	1 B
4. What ethical and legal framework can support healthcare providers in decisions about the use of physical restraint in home care?	7.	Consider carefully the different values, norms and reasons in the context of humane care.	1 C
	8.	Physical restraints may only be used as a last resort and exception. A clear reporting of the careful decision-making process in the patient record is necessary.	1 *
		* No strength of evidence because it is based on legal texts	
5. How can healthcare workers reduce physical restraint use in home care?	9.	Healthcare providers should reduce restraint use in home care. The following elements should be considered:	1 B
		1. Gain insight into personal and contextual factors: thorough assessment	
		2, Collaborate with interdisciplinary team (including patient and family) and take personal responsibility.	
		3. Communicatie proactively and transparently with all involved persons.	
		4. Develop a care plan with the involved persons (formal and informal caregivers) to determine the aims and preventive actions.	
6. What steps and persons need to be involved in the decision-making process regarding and the application of physical restraints in home care? (see flowchart Fig. 1)	10.	A successful decision-making process to reduce physical restraints in home care should consists of the following components:	1 C
		- carefully and consciously dealing with situations where means of physical restraints are considered, requested or already used;	
		- taking the preferences of the patient into account;	
		- involving the patient and the family and all other involved healthcare providers from the beginning of the process.	
		Physical restraint is a last resort and should only be used after first considering alternatives, over a short period of time, with careful supervision and with materials that are in proportion to the patient’s behaviour.	

GRADE: The strength of the recommendation is based on the GRADE methodology expressed in a number (1 = strong; 2 = weak). The quality of the evidence is classified into high (A), moderate (B) or low (C) (Van Royen et al., 2008 [34])

### What is meant by physical restraint use in home care?

Given the absence of a clear consensus on the definition of physical restraints when the guideline was developed, the definition of Retsas (1998) [45] was used [recommendation 1]. This comprehensive definition is widely used in the residential setting and seems to be useful for home care. The use of psychoactive drug, electronic supervision or prescribed orthopaedic devices that are part of a treatment process are not considered physical restraints. Examples of physical restraints in home care are bedrails, bed-against-the-wall (positioned in a way that the person will not fall out of bed), locked room or house doors, deep chair that prevents rising, and restrictive clothing and belts [3].

### What factors affect the probability of physical restraints being used in home care?

Physical restraints were often considered by nurses and informal caregivers to be safety measures [10]. For this reason, the second recommendation of the guideline emphasized that healthcare providers must be aware that the application of any measure that limits the free movement of the patient – regardless of its purpose – is a form of restraint because of the possible negative impact of its use and the lived experience of the patient (see question 3) [recommendation 2]. A combination of factors influences the use of physical restraints. Person-related factors (e.g., cognitive impairment, poor mobility, dependency in activities of daily living, and challenging behaviour) are the main predictive factors. In addition, context-related factors (e.g., the family frequently asking healthcare staff to use restraints, the wellbeing of the informal caregiver, and informal caregiver dissatisfaction with family support) [12] and factors related to healthcare providers and their organization (e.g., lack of awareness and knowledge of the negative impact of restraint use by healthcare providers, lack of staff knowledge regarding person-centred care, behavioural communication and ways to meet patient needs and avoid behaviours that precipitate a caregiver wish for restraint, and a lack of clear policy regarding restraint use within the organization) influence physical restraint use [14, 41]. Furthermore, legislative (e.g., who is allowed to use physical restraints, and regulation re: informed consent) and policy-related issues also influence restraint use. [recommendation 3] [14, 41]. In home care, in addition to the above-mentioned factors, supervision, informal caregivers’ decreased wellbeing and dissatisfaction with family support are also associated with restraint use [10, 11].

### What are the consequences and the impact of the use of physical restraints in home care?

The overview of the negative consequences of the use of physical restraints is based primarily on evidence from the literature on residential care settings (mainly from nursing homes) and considers the patient, the family and the healthcare provider. Patients can experience physical consequences such as pressure injuries, urinary incontinence, constipation, increased risk of falls, increased dependence in activities of daily living and decreased muscle strength and mobility [14, 25, 41, 42, 46]. In addition to the negative physical consequences, restraint use affects patient psychosocial wellbeing (e.g., it can result in depression, social isolation, discomfort, indifference, fear, anger, and humiliation) [14, 26, 41, 43].

Informal caregivers (e.g., family members and relatives) are important for the continuity of home care and play an important role in the use of restraints. Depending on the home care situation and the level of involvement, the impacts of physical restraint use on the experiences and psychosocial wellbeing of informal caregivers may differ (positively or negatively) [1, 10, 11, 47]. Limited research indicates that some family members associate the use of physical restraints with finality associated with live and existence and emotions such as anger and disillusionment [14].

Physical restraint use also involves healthcare workers. Indeed, considerations of physical restraint use are complex decision-making processes influenced by patient-, nurse- and context-related factors [15]. The factors specific to the home care setting (e.g., the important influencing role of the informal caregiver in the process of caregiving) implies that healthcare providers are often faced with difficult home care situations and decisions that affect their experiences and emotional wellbeing (e.g., the mixed emotions of anger and guilt over having to apply restraints and experiencing relief from the benefits of applying the restraints) [10, 14, 19, 47].

The important impact of restraint use on all involved persons has resulted in the recommendations that the use of physical restraint be avoided as much as possible [recommendation 4] and that healthcare providers [recommendation 5] and healthcare organizations [recommendation 6] be aware of the consequences of their use.

### Which ethical and legal framework can support healthcare providers in decisions about the use of physical restraint in home care?

Reduction of physical restraint use requires a critical and thorough consideration and weighing of values, norms and reasons within the framework of human, person-centred care [14, 15, 43, 48]. Based on the literature, the following four important values are highlighted in the guideline: respect for the dignity of older persons, autonomy, self-reliance and overall wellbeing [recommendation 7].

Although there is no specific legislation pertaining to physical restraint use in Belgium, some legal principles need to be respected and are included in the guideline. In general, the use of physical restraint is not allowed (i.e., the freedom of a person is guaranteed according to Article 5 of Belgian Law, and the right to freedom and safety of the person is mentioned in Article 5 of the European Convention for the Protection of Human Rights). An exception for physical restraint use can be made, provided that legal considerations are respected and there is strict compliance with the existing regulations. In Belgium, only nurses and physicians are allowed to apply physical restraints in the context of providing healthcare. The guideline integrates the patient’s rights (e.g., informed consent for persons who are competent to make decisions and proxy consent for persons who are incapable of giving informed permission) [35, 36, 50]. The guideline emphasizes the need for the utmost care (i.e., the application of the carefulness principle) when the use of physical restraints is considered, requested or already present. Due care implies that healthcare providers clearly report in the patient record all steps of the decision-making process to ensure high standards of care. Important aspects of this decision-making process include organizing a meeting with all involved persons; discussing the underlying behaviour or circumstances resulting in the use of physical restraints; and comprehensively reporting, using relevant documentation, the decision-making, application and evaluation processes. All healthcare providers involved in the decision must be informed. The decision must then be carefully and correctly implemented, and follow-up measures must be taken. The guideline stipulates that the ethical and professional responsibility of the decision lies equally with all persons involved. Based on legal principles, the guideline requires that physical restraints be used only as a last resort [recommendation 8].

### How can healthcare workers reduce restraint use in home care?

The guideline describes interventions other than restraints that can be considered for each of the four types of risks/behaviours (i.e., a fall; agitation and confusion; wandering; and delirious behaviour) that often lead to the unjustified use of (physical) restraints. For each risk behaviour, the alternatives are subdivided into evaluating and reviewing possible medical or physiologic causes (e.g., polypharmacy, pain/discomfort, hunger, and full bladder); optimizing the environment (e.g., removing physical obstacles); alternatives in and around the bed (e.g., a height-adjustable bed); supporting the person (e.g., encouraging physical movement and introducing pleasurable activities that match the patient’s preferences, interests and abilities); support in walking (e.g., supportive shoes, walking frame); and transfer in or out of the bed, seat or chair.

In developing more appropriate interventions, it is important that all involved healthcare workers, older persons and informal caregivers be involved in the decision-making process regarding restraint, with the goal of avoiding, minimizing or discontinuing the use [23, 26, 28, 39, 42].

### What steps and persons need to be involved in the decision-making process regarding and the application of physical restraints in home care? (Fig. 1)

The complexity of restraint use in home care requires a careful and deliberate process of dealing with situations in which restraints are considered, requested or already in use, with the goal of keeping the use as low as possible. Minimizing restraint use requires critical analysis of the reasons leading to restraint use and, at the same time, a search for preventive actions and more appropriate interventions.

**Fig. 1:**
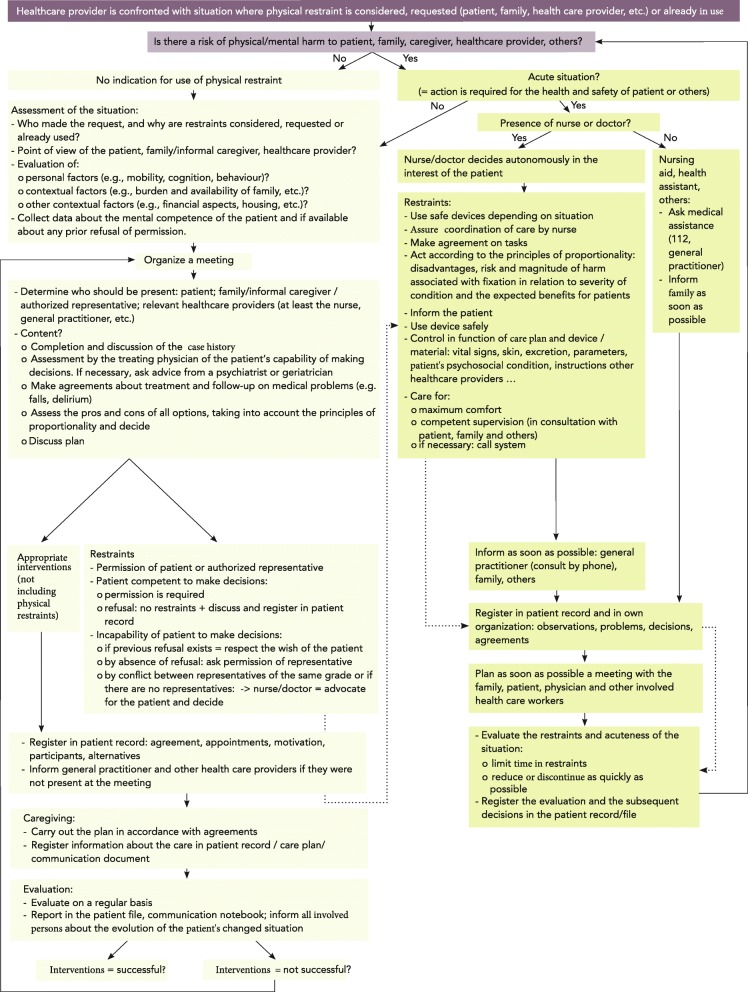
Flowchart

A comprehensive assessment of the person- and context-related factors will lead to a better understanding of the patient and the home environment. These insights are the basis for developing an individual care plan for the patient, through the cooperation of all involved clinicians, the patient and informal caregivers. Other key elements include proactive and transparent communication and the commitment that each involved person has to fulfil his or her responsibilities (e.g., in accordance with the agreements made) [23, 26, 28, 39, 42] [recommendation 9].

The patient and the family, together with all involved healthcare workers, must be included in the decision-making process regarding restraint use and the discussion of alternative or more appropriate interventions from the beginning. The various means of meeting the patients’ needs must be considered first; any use of restraint is always a last and temporary resort. If continued restraint is deemed necessary, then the device or method used should be least restrictive given the patient’s behaviour, increased supervision should be implemented and the use of restraints should be stopped as soon as possible [14, 26] [recommendation 10].

A flowchart has been developed (Fig. 1) to support healthcare providers in minimizing the use of physical restraint. This flowchart divides the decision-making process into a number of steps to guide healthcare providers through complex home care situations where restraints are requested or already used, and alternatives are discussed.

The first step consists of an evaluation of the individual patient situation, i.e., whether the patient’s behaviour poses a physical risk and/or mental harm to the patient or others. If there is no risk of harm, physical restraints may not be used. If there is a risk for harm, a distinction should be made between urgent and non-urgent situations.

Urgent situations require immediate action be taken in the interests of the patient’s health and safety and that of others. In assessing a patient’s behaviour and responding to meet the need expressed, nurses and physicians act autonomously and can decide to apply physical restraints. Other healthcare providers (e.g., those not legally authorized to use physical restraints according to the Belgian law) ask for medical assistance (e.g., by using an emergency call) and immediately inform the patient’s general practitioner and family. A meeting between the patient, the family, the general practitioner and other healthcare workers involved must be planned to re-assess the urgency of the patient’s situation and the necessity of restraint use to limit the use of restraints over time and as quickly as possible.

In a non-urgent situation, information on patient- and context-related factors needs to be collected and discussed with all involved persons. The general practitioner needs to assess the capability of the patient to make decisions, consulting with the multidisciplinary team on this point as needed. The pros and cons of all options to manage the home care situation need to be assessed and discussed. Considering that the use of restraints is almost never the first choice, the team jointly decides with the patient and the informal caregiver which option (use of restraints, more appropriate interventions or a combination of both) is most suitable. If physical restraint use is considered, the team must ensure that the benefits of the restraint use outweigh the associated risks and that the physical restraint is stopped as soon as possible and used solely for the interest and wellbeing of the patient. The permission of the patient, or his/her representative in the case of a person who is incapable of making decisions, is required before applying physical restraints.

The decision-making process, the caregiving and regular evaluations must always be reported in the patient record, and all involved persons must be informed.

## Discussion

Current evidence indicates that restraints are regularly used in home care; that they are mainly applied to vulnerable older persons; and that informal caregivers, who have less knowledge of the negative consequences of restraint use, play a prominent role in the application of restraints, by granting permission for the use of restraints and deciding to use restraints [47, 48]. Nurses also play a pivotal role in the use of restraints, and as recent studies suggest, they have insufficient knowledge of the concept of restraints, their frequent use in clinical practice and the negative impact on the patient [9]. The complexity of the issue of restraint use in home care is made clear by these findings combined with the lack of a clear definition, the ethical dilemmas facing healthcare workers regarding using physical restraints, the current legal framework failing to provide clear guidance for clinical practice in Belgian home care, and the lack of clear policy [14, 28]. There is an urgent need for a practice guideline to support home care clinicians in properly managing the restraint use dilemma. To the best of our knowledge, this is the first validated evidence-based practice guideline for reducing the use of physical restraint in home care. Considering the different aspects that contribute to the complexity of restraint use in home care, the guideline was developed by a multidisciplinary workgroup.

The current guideline aims to support healthcare workers in reducing the use of physical restraints and optimizing the quality of care for older adults receiving home care.

To ensure a deliberate and careful approach to limiting or preventing physical restraint use in home care, our guideline focuses on increasing knowledge and awareness by providing information on this topic (Question 1–5), which is necessary for understanding and implementing the flowchart (Question 6). The knowledge obtained through the answers to the different questions and the flowchart aim to stimulate a deliberate and careful decision-making process related to restraint use in home care.

When using this guideline, the following points should be taken into account. The guideline is not a protocol or standing order but a tool to support professional healthcare providers dealing with situations where restraints are requested or already in place. The guideline explicitly aims to support healthcare providers in reducing restraint use in aged community care by increasing home health care providers’ awareness, knowledge and competence regarding restraint use. More concretely, the guideline provides information about the concept and the negative impact and consequences of restraint use in home care as well as a flowchart to support a careful and well-considered decision-making process in practice. Healthcare providers must ensure that the use of restraint use is always a last and temporary resort.

The guideline integrates Belgian legislation on restraint use with patient rights legislation. A limited literature search suggests that, despite international differences in legislation, the European Convention on Human Rights (2010) is (mainly) used as a guiding principle for dealing with restraint use (i.e., informed and voluntary treatment).

Evidence on restraint use in home care is scarce. For this reason, the multidisciplinary working group translated, adapted and used the strong evidence on restraint use in the residential setting (mainly nursing homes). The resulting recommendations in the current guideline were achieved through consensus. More research on specific issues, such as the experiences of the patient, family and other involved healthcare providers in home care and in the use of patient-centred interventions rather than restraints in home care, is necessary.

Insufficient time and resources precluded us from conducting a systematic literature review including all types of empirical studies. A recently published systematic review [4], however, did not reveal additional studies, confirming the scarcity of literature on restraint use in home care. Studies published after completion of the systematic review [5, 47], did not change the content of the guideline; and as a consequence, all statements and recommendations of the guideline still stand based on the best available evidence to date.

The full guideline has been published in a Dutch book format [33]. The attachments (i.e., literature search and tables of quality assessments) are available on the website of the publisher and can be consulted through a code included in the book. A last limitation is the fact that the guideline is not available free of charge.

The guideline was validated by an independent organization, e.g., the Belgian Centre for Evidence-Based Medicine (CEBAM), which guarantees the methodological quality of the practice guideline. Thorough testing of its acceptability and usability in the field, however is necessary. Our follow-up project, which aims to develop and evaluate a multicomponent programme to implement the practice guideline, will provide this testing.

Finally, the development of the practice guideline focused solely on the use of ‘physical’ restraints as defined by Retsas (1998) [45]. The new international definition of physical restraints [37] became available only after the guideline was finalized. Previous research shows that restraint use in home care can involve more than just “physical” restraints (e.g., adaptation of the house, forced or camouflaged medication administration, and locking the house) [3, 4, 9, 33]. A recent systematic review indicates that there is still a lack of a clear conceptualization of restraint use in home care [4]. This review revealed different terms and definitions (i.e., ‘restraints’, ‘physical restraints’ and ‘involuntary treatment’) associated with the concept of restraints. All of these definitions include some appeal to physical restraint, emphasize the intentional and deliberate restriction of a person’s voluntary movement and refer to the impact on the involved person of its application [9]. Having a clear definition that takes into account the specificity of the home care setting will help increase awareness among healthcare providers and be a first step in reducing the use of restraints in this setting [9]. Therefore, the definition of restraint use should be clarified by the next update of the guideline.

## Practical implications

The availability of an evidence-based guideline is an essential first step towards a reduction of restraint use in home care. Next, the current guideline needs to be implemented in practice, and the effect of this implementation needs to be evaluated. Evidence from the residential care setting shows that guidelines alone are insufficient to reduce restraint use. Furthermore, the success of reducing physical restraint use in the long term depends on a multicomponent strategy that involves policy change, leadership, education, and expert consultation [38, 51, 52]. To reduce the use of physical restraints, it is important, among other things, that healthcare providers be sensitized to the impact and consequences on patients and that organizations facilitate the implementation of the guideline. This requires additional investment at various levels (e.g., availability of alternatives; a clear policy on restraint use; organization of regular multidisciplinary meetings; and investment in the education of healthcare providers, with specific attention on supporting informal caregivers).

Therefore, the authors developed a new multicomponent programme to implement the guideline in clinical practice. By using the six steps of intervention mapping, a multicomponent programme was developed in a systematic manner in conjunction with an expert group of stakeholders. Various implementation aids are available for free on the website www.fixatiearmezorg.be. Eight components were developed: a summary of the guideline, a flyer to promote communication with informal caregivers, a website, social media (i.e., Facebook and twitter), a promo film, a physical restraint checklist, 2 online tutorials and an ‘ambassador for restraint-free home care’ training programme. The programme was tested in a pilot study and evaluated using a mixed methods design [53].

## Conclusion

The current guideline is the first validated, evidence-based guideline for reducing the use of physical restraints at home. Due to the limited but recent evidence available in the home care setting, evidence from the residential setting was also used to develop this guideline. This evidence was translated to the home care setting according to a consensus procedure using a multidisciplinary working group. Representatives of patients and informal caregivers were among the members of the multidisciplinary working group. An updated guideline that includes the perspectives of patients and informal caregivers is therefore necessary. This updated guideline should be pilot tested in real home care situations, be made available in English, and be made widely accessible. Further investments in its implementation will demonstrate the potential of the current guideline. Considering the complexity of the home care setting, there is an urgent need for effective multidisciplinary collaboration and further research.

## Supplementary information

**Additional file 1: Figure S1.** Overview of all searches per clinical question (home care and residential setting).

## Data Availability

Not applicable.

## Abbreviations

Agree II: Appraisal of Guidelines for Research & Evaluation II
AMSTAR: Assessment of multiple systematic reviews
CEBAM: Belgian Centre for Evidence-Based Medicine
GRADE: Grading of Recommendations Assessment, Development and Evaluation
KCE: Federaal kenniscentrum voor Gezondheidszorg
MINORS: Methodological Index for Non-Randomized Studies
NICE: National Institute for Health and Care Excellence
SING: Scottish Intercollegiate Guidelines Network
VAKS: Danish acronym for Appraisal of Qualitative Studies
WHO guidelines: World Health Organization guidelines
