# Supplementary material for: Reducing physical restraints by older adults in home care: development of an evidence-based guideline
Source: BMC Geriatr. 2020 May 7;20:169. doi: 10.1186/s12877-020-1499-y (PMC7204038; doi:10.1186/s12877-020-1499-y)
Supplement: Supplementary file 1 — Additional file 1: Figure S1. Overview of all searches per clinical question (home care and residential setting). [file 12877_2020_1499_MOESM1_ESM.docx]

**Overview of all search strategies per clinical practice question (home care and residential setting)**

**Clinical practice question 1:** What is meant by physical restraints in home care?

Home care

Articles included after full text revisions (n = 7)

Reason exclusion: 1 article not available

Reasons for exclusion of articles based on screening of title and abstract (n= 383):

- Setting (n = 108)
- Design (n = 3)
- Study aim (n = 262)
- Language (n = 10)

Articles after exclusion based on screening of title and abstract (n = 8)

Database after exclusion of duplicate articles (n = 391)

Databases (n= 470):

- Pubmed (n = 120)
- Cinahl (n = 30)
- Embase (n = 320)

Total: n= 470

Search terms used:

- Immobilization OR physical restraint OR physical restraints OR "Restraint, Physical" OR restraints

AND

- "Aged" OR "Frail Elderly" OR oldest old OR elders OR elder OR Aged, 80 and Over

AND

- "Home Care Services" OR "Home Care Services" OR "Home Care Service" OR "Domiciliary Care" OR "home care" OR "home care agencies" OR "home health agency" OR "home health agencies" OR "home health care agency" OR "home health care agencies" OR "Primary Health Care" OR "Primary Health Care" OR "Primary HealthCare" OR "Primary Care" OR "Primary Care Nursing" OR "Primary Care Nursing" OR "Home Nursing" OR "Home Nursing" OR "community care" OR “community health” OR “community health service” OR “community health services” OR ”community health care” OR ”community healthcare” OR "nurses, community health" OR "Community Health Nurse" OR "Community Health Nurses" OR "home nurse" OR "home nurses" OR "home health nurses" OR "home health nurse" OR "Home Care Services, Hospital-Based” OR "home health service" OR "home health services"

**Clinical practice question 2:** What factors affect the probability of physical restraints in home care?

Articles included after full text revisions (n = 4)

Reason exclusion: 2 articles over the same study were taken together

Articles after exclusion based on screening of title and abstract (n = 5)

Reasons for exclusion of articles based on screening of title and abstract (n = 184):

- Setting (n = 57)
- Design (n = 8)
- Study aim (n = 114)
- Language (n = 5)

Database after exclusion of duplicate articles (n = 189)

Filter: Publications of (systematic) reviews during the last 5 years

Databases (n = 203):

- Pubmed (n = 138)
- Cinahl (n = 65)

Search terms used:

- immobilization OR physical restraint OR physical restraints OR "Restraint, Physical" OR restraints

AND

- "Aged" OR "Frail Elderly" OR oldest old OR elders OR elder OR Aged, 80 and Over

AND

- (review of the literature) OR systematic review) OR ("Review Literature as Topic OR "Review)

Residential setting (mainly nursing homes)

Home care

Articles included after full text revisions (n = 1)

Reasons for exclusion of articles based on screening of title and abstract (n = 43):

- Setting (n = 12)
- Study Aim (n = 26)
- Language (n = 5)

Articles after exclusion based on screening of title and abstract ( n = 1)

Database after exclusion of duplicate articles (n = 44)

Databases (n = 51):

- Pubmed (n = 37)
- Cinahl (n = 14)

Search terms used:

- Immobilization OR physical restraint OR physical restraints OR "Restraint, Physical" OR restraints

AND

- "Aged" OR "Frail Elderly" OR oldest old OR elders OR elder OR Aged, 80 and Over

AND

- "Home Care Services" OR "Home Care Services" OR "Home Care Service" OR "Domiciliary Care" OR "home care" OR "home care agencies" OR "home health agency" OR "home health agencies" OR "home health care agency" OR "home health care agencies" OR "Primary Health Care" OR "Primary Health Care" OR "Primary HealthCare" OR "Primary Care" OR "Primary Care Nursing" OR "Primary Care Nursing" OR "Home Nursing" OR "Home Nursing" OR "community care" OR “community health” OR “community health service” OR “community health services” OR ”community health care” OR ”community healthcare” OR "nurses, community health" OR "Community Health Nurse" OR "Community Health Nurses" OR "home nurse" OR "home nurses" OR "home health nurses" OR "home health nurse" OR "Home Care Services, Hospital-Based” OR "home health service" OR "home health services"

AND

- “Risk Factors" OR "Risk Factors" OR "causality" OR "causality" OR ("predisposing" AND "factors") OR "predisposing factors" OR ("Causality" AND "etiology") OR characteristics

Articles included after full text revisions (n = 2)

Articles after exclusion based on screening of title and abstract (n = 2)

Database after exclusion of duplicate articles (n = 54)

Databases (n = 57):

- Pubmed (n = 26)
- Cinahl (n = 31)

Filter: Publications of (systematic) reviews during the last 5 years

Reasons for exclusion of articles based on screening of title and abstract (n = 52):

- Setting (n = 12)
- Study aim (n = 34)
- Design (n = 6)

Search terms used:

- Immobilization OR physical restraint OR physical restraints OR "Restraint, Physical" OR restraints

AND

- "Aged" OR "Frail Elderly" OR oldest old OR elders OR elder OR Aged, 80 and Over

AND

- (review of the literature) OR (systematic review) OR ("Review Literature as Topic OR "Review)

AND

- “Risk Factors" OR "Risk Factors" OR "causality" OR "causality" OR ("predisposing" AND "factors") OR "predisposing factors" OR ("Causality" AND "etiology") OR characteristics

Residential setting (mainly nursing homes)

**Clinical practice question** 3: What are the consequences and the impact of the use of physical restraint in home care?

Articles included after full text revisions (n = 2)

Articles after exclusion based on screening of title and abstract (n = 2)

Reasons for exclusion of articles based on screening of title and abstract (n= 46):

- Setting (n = 13)
- Study aim (n = 28)
- Language (n = 5)

Database after exclusion of duplicate articles (n = 48)

Databases (n= 53):

- Pubmed (n = 45)
- Cinahl (n = 8)

Home care

Search terms used:

- immobilization OR physical restraint OR physical restraints OR "Restraint, Physical" OR restraints

AND

- "Aged" OR "Frail Elderly" OR oldest old OR elders OR elder OR Aged, 80 and Over

AND

- "Home Care Services" OR "Home Care Services" OR "Home Care Service" OR "Domiciliary Care" OR "home care" OR "home care agencies" OR "home health agency" OR "home health agencies" OR "home health care agency" OR "home health care agencies" OR "Primary Health Care" OR "Primary Health Care" OR "Primary HealthCare" OR "Primary Care" OR "Primary Care Nursing" OR "Primary Care Nursing" OR "Home Nursing" OR "Home Nursing" OR "community care" OR “community health” OR “community health service” OR “community health services” OR ”community health care” OR ”community healthcare” OR "nurses, community health" OR "Community Health Nurse" OR "Community Health Nurses" OR "home nurse" OR "home nurses" OR "home health nurses" OR "home health nurse" OR "Home Care Services, Hospital-Based” OR "home health service" OR "home health services"

AND

- Adverse effects OR impact OR consequences OR injury

Articles included after full text revisions (n = 3)

Reasons for exclusion of articles based on screening of title and abstract (n = 44):

- Setting (n = 12)
- Design (n = 29)
- Study aim (n = 3)

Articles after exclusion based on screening of title and abstract (n = 3)

Database after exclusion of duplicate articles (n = 47)

Databases (n= 49):

- Pubmed (n = 32)
- Cinahl (n = 17)

Totaal: n= 203

Filter: Publications of (systematic) reviews during the last 5 years

Search terms used:

- immobilization OR physical restraint OR physical restraints OR "Restraint, Physical" OR restraints

AND

- "Aged" OR "Frail Elderly" OR oldest old OR elders OR elder OR Aged, 80 and Over

AND

- (review of the literature) OR systematic review) OR ("Review Literature as Topic OR "Review)

AND

- adverse effects OR impact OR consequences OR injury

Residential setting (mainly nursing homes)

**Clinical practice question 4:** What ethical and legal framework can support healthcare providers in decisions about the use of physical restraint in home care?

Home care

Search terms used:

- Codes of Ethics OR "Principle-Based Ethics" OR "Ethics, Professional" OR "Ethics, Nursing" OR "ethics" OR "Ethics, Clinical" OR "Ethics" OR “decision making” OR "decision "AND "making"

AND

- Immobilization OR physical restraint OR physical restraints OR "Restraint, Physical" OR restraints

AND

- "Aged" OR "Frail Elderly" OR oldest old OR elders OR elder OR Aged, 80 and Over

AND

- "Home Care Services" OR "Home Care Services" OR "Home Care Service" OR "Domiciliary Care" OR "home care" OR "home care agencies" OR "home health agency" OR "home health agencies" OR "home health care agency" OR "home health care agencies" OR "Primary Health Care" OR "Primary Health Care" OR "Primary HealthCare" OR "Primary Care" OR "Primary Care Nursing" OR "Primary Care Nursing" OR "Home Nursing" OR "Home Nursing" OR "community care" OR “community health” OR “community health service” OR “community health services” OR ”community health care” OR ”community healthcare” OR "nurses, community health" OR "Community Health Nurse" OR "Community Health Nurses" OR "home nurse" OR "home nurses" OR "home health nurses" OR "home health nurse" OR "Home Care Services, Hospital-Based” OR "home health service" OR "home health services"

Databases (n= 43):

- Pubmed (n = 3)
- Cinahl (n = 40)

Reasons for exclusion of articles based on screening of title and abstract (n= 43)

- Setting (n = 9)
- Design (n = 1)
- Study aim (n = 26)
- Language (n = 1)
- Not available (n = 6)

Database after exclusion of duplicate articles (n = 43)

Articles after exclusion based on screening of title and abstract (n = 0)

Search terms used:

- Codes of Ethics OR "Principle-Based Ethics" OR "Ethics, Professional" OR "Ethics, Nursing" OR "ethics" OR "Ethics, Clinical" OR "Ethics" OR “decision making” OR "decision "AND "making"

AND

- immobilization OR physical restraint OR physical restraints OR "Restraint, Physical" OR restraints

AND

- "Aged" OR "Frail Elderly" OR oldest old OR elders OR elder OR Aged, 80 and Over

AND

- (review of the literature) OR systematic review) OR ("Review Literature as Topic OR "Review)

Residential setting (mainly nursing homes)

Filter: Publications of (systematic) reviews during the last 5 years

Articles included after full text revisions (n = 2)

Articles after exclusion based on screening of title and abstract (n = 2)

Reasons for exclusion of articles based on screening of title and abstract (n= 63) :

- Setting (n = 13)
- Design (n = 6)
- Study aim (n = 42)
- Outcome (n =2)

Database after exclusion of duplicate articles (n = 65)

Databases (n= 67):

- Pubmed (n = 3)
- Cinahl (n = 64)

**Clinical practice question 5 & 6:** How can healthcare workers reduce restraints in home care and what steps and which persons need to be involved in the decision-making process and the application of physical restraints in home care?

Home care

Search terms used:

- alternatives OR intervention OR nursing intervention OR reduction OR prevention OR education program OR "Education" OR restraint free care OR "alternative" OR "alternatives"

AND

- immobilization OR physical restraint OR physical restraints OR "Restraint, Physical" OR restraints

AND

- "Aged" OR "Frail Elderly" OR oldest old OR elders OR elder OR Aged, 80 and Over

AND

- "Home Care Services" OR "Home Care Services" OR "Home Care Service" OR "Domiciliary Care" OR "home care" OR "home care agencies" OR "home health agency" OR "home health agencies" OR "home health care agency" OR "home health care agencies" OR "Primary Health Care" OR "Primary Health Care" OR "Primary HealthCare" OR "Primary Care" OR "Primary Care Nursing" OR "Primary Care Nursing" OR "Home Nursing" OR "Home Nursing" OR "community care" OR “community health” OR “community health service” OR “community health services” OR ”community health care” OR ”community healthcare” OR "nurses, community health" OR "Community Health Nurse" OR "Community Health Nurses" OR "home nurse" OR "home nurses" OR "home health nurses" OR "home health nurse" OR "Home Care Services, Hospital-Based” OR "home health service" OR "home health services"

Databases (n= 55):

- Pubmed (n = 44)
- Cinahl (n = 11)

Database after exclusion of duplicate articles (n = 51)

Reasons for exclusion of articles based on screening of title and abstract (n= 48):

- Setting (n = 22)
- Study aim (n = 24)
- Language (n = 1)
- Not available (n = 1)

Articles after exclusion based on screening of title and abstract (n = 3)

Articles included after full text revisions (n = 3)

Filter: publicaties van (systematische) reviews gedurende de laatste 5 jaar

Reden exclusie:

- Setting: n = 4
- Design: n = 5
- Doel studie: n = 31
- Outcome: n = 1

Reden exclusie:

- Outcome: n = 1 – zelfde studies

Weerhouden: n = 1

Na lezen van titel en abstract: n = 2

Pubmed (n = 24)| Cinahl (n = 22)

Totaal: n = 46

Na controle op dubbele artikels: n = 43

Filter: Publications of (systematic) reviews during the last 5 years

Search terms used:

- alternatives OR intervention OR nursing intervention OR reduction OR prevention OR education program OR "Education" OR restraint free care OR "alternative" OR "alternatives"

AND

- immobilization OR physical restraint OR physical restraints OR "Restraint, Physical" OR restraints

AND

- "Aged" OR "Frail Elderly" OR oldest old OR elders OR elder OR Aged, 80 and Over

AND

- (review of the literature) OR systematic review) OR ("Review Literature as Topic OR "Review)

Residential setting (mainly nursing homes)

Databases (n= 46):

- Pubmed (n = 24)
- Cinahl (n = 22)

Reason exclusion: Outcome (n = 1) – same study

Articles included after full text revisions (n= 1)

Articles after exclusion based on screening of title and abstract (n = 2)

Reasons for exclusion of articles based on screening of title and abstract (n = 41) :

- Setting (n = 4)
- Design (n = 5)
- Study aim (n = 31)
- Outcome (n =1)

Database after exclusion of duplicate articles (n = 43)
